# Prevalence and Clinical Impact of Concomitant Mutations in Anaplastic Lymphoma Kinase Rearrangement Advanced Non-small-Cell Lung Cancer (Guangdong Association of Thoracic Oncology Study 1055)

**DOI:** 10.3389/fonc.2020.01216

**Published:** 2020-08-21

**Authors:** Meichen Li, Xue Hou, Chengzhi Zhou, Weineng Feng, Guanming Jiang, Hao Long, Shuang Yang, Jing Chen, Na Wang, Kaicheng Wang, Likun Chen

**Affiliations:** ^1^Department of Medical Oncology, Sun Yat-sen University Cancer Center, State Key Laboratory of Oncology in South China, Collaborative Innovation Center for Cancer Medicine, Guangzhou, China; ^2^State Key Laboratory of Respiratory Disease, National Clinical Research Center for Respiratory Disease, Guangzhou Institute of Respiratory Disease, The First Affiliated Hospital of Guangzhou Medical University, Guangzhou, China; ^3^Department of Head and Neck/Thoracic Medical Oncology, The First People's Hospital of Foshan, Foshan, China; ^4^Dongguan People's Hospital, Dongguan, China; ^5^Department of Thoracic Surgery, Sun Yat-sen University Cancer Center, Guangzhou, China

**Keywords:** *ALK* rearrangement, non–small-cell lung cancer, concomitant mutations, crizotinib, next-generation sequencing

## Abstract

**Background:** In patients with anaplastic lymphoma kinase *(ALK*) rearrangement-positive advanced non–small-cell lung cancer (NSCLC), *ALK* inhibitors are now the standard treatment, but their clinical efficacy varies widely for each patient. In this multicenter retrospective study, we evaluated the clinical efficacy of crizotinib according to the *ALK* rearrangement variants and concomitant mutations present.

**Patients and Methods:** A total 132 patients with *ALK* rearrangement advanced NSCLC from 4 centers in Guangdong province, China were evaluated. All patients received crizotinib treatment and their *ALK* rearrangement status was identified by next-generation sequencing (NGS).

**Results:** The median progression-free survival (PFS) in patients with EML4-*ALK* rearrangement (*n* = 121), non-EML4-*ALK* rearrangement (*n* = 5), and EML4-*ALK* arrangement accompanied by non-EML4-*ALK* rearrangement (*n* = 6) was 12.8, 7.5, and 7.4 months, respectively, with no significant difference between them (*p* = 0.1554). Similarly, among patients with various EML4-*ALK* variants (variant 1, variant 3a/b, and other variants), the median PFS values were again comparable. According to baseline NGS data, the median PFS in patients who had *ALK* rearrangement only, *ALK* rearrangement and concomitant tumor-suppressor gene mutations, and *ALK* rearrangement and concomitant oncogene mutations was 14.2, 10.9, and 4.9 months, respectively; (*p* = 0.0002). A multivariable analysis indicated that concomitant oncogene mutations and tumor-suppressor gene mutations were both negative factors influencing the efficacy of crizotinib in *ALK* rearrangement NSCLC.

**Conclusion:** Concomitant oncogene mutations and tumor-suppressor gene mutations had negative effects on the efficacy of crizotinib, while various *ALK* variants had a similar influence.

## Introduction

Lung cancer remains the leading cause of cancer deaths in China. In patients with non–small-cell lung cancer (NSCLC), anaplastic lymphoma kinase (*ALK*) gene rearrangement is detected in approximately 3–7% of cases (1). In 2007, Soda et al. (2) first identified the echinoderm microtubule-associated protein-like 4 *(EML4*)-*ALK* fusion oncogene in NSCLC. Currently, more than 20 *ALK* rearrangement variants have been discovered, the most frequent among which are variant 1 (E13:A20) and variant 3a/b (E6a/b:A20) (3). All variants contain the *ALK* tyrosine kinase domain and an oligomerization domain in the N-terminal fusion partner gene, which activate downstream pathways to control the proliferation and apoptosis of carcinoma cells. In addition, more non-*EML4* fusion variants have been discovered, including kinesin family member 5B (*KIF5B*) (4), kinesin light-chain 1 (*KLC1*) (5), cut-like homeobox 1 gene (*CUX1*) (6). Huntingtin-interacting protein 1(*H1P1*) (7), translocated promoter region (*TPR*) (8), baculoviral inhibition of apoptosis protein repeat-containing 6 (*BIRC6*) (9), and S1 RNA binding domain 1 (*SRBD1*) (10). These variants have all shown clinical responses to *ALK* inhibitors.

Since the first-generation *ALK* tyrosine kinase inhibitor (TKI) crizotinib (11, 12) was introduced, the development of targeted therapy has greatly improved the survival time and quality-of- life of patients with *ALK* rearrangement advanced NSCLC. In addition, second- and third-generation *ALK* TKIs, including ceritinib (13), alectinib (14), brigatinib (15), and lorlatinib (16), have also shown significant efficacy in these patients. However, despite their efficacy in patients with *ALK* rearrangement, all patients inevitably develop resistance to treatment and clinical efficacy varies widely for each patient. To date, a series of studies have investigated whether different *ALK* variants may affect the clinical response in patients who receive *ALK* inhibitors, and whether they are associated with resistance mechanisms. Lin et al. (17) have previously reported that *ALK* G1202R is significantly more common with variant 3 than variant 1 (57 vs. 30%; *p* = 0.023).

With the rapid development of next-generation sequencing (NGS), more and more *ALK* concomitant genes have been found. Epidermal growth factor receptor (*EGFR*) mutations are the most common mutations in NSCLC, there have been a series of studies showing that concomitant mutations are associated with inferior efficacy of *EGFR* TKI therapy (18, 19). In *ALK* rearrangement advanced NSCLC, it is still unclear whether concomitant mutations are negative predictive factors for *ALK* TKI therapy. Some retrospective studies and case reports have reported the poor efficacy of crizotinib treatment for *ALK* rearrangement NSCLC co-occurring with *TP53, KRAS* and *EGFR* mutations (20, 21). Therefore, we performed a retrospective multicenter study to explore the factors affecting the efficacy of crizotinib according to baseline next-generation sequencing data in patients with *ALK* rearrangement*-*positive advanced NSCLC.

## Materials and Methods

### Patients

Between January 2012 and June 2019, a total of 132 patients with *ALK* rearrangement advanced NSCLC from 4 medical centers across Guangdong province, China were evaluated. All patients had been histologically diagnosed with NSCLC, and with clinical stage IIIB, IV or recurrent disease according to the 7th American Joint Committee on Cancer (AJCC) staging system. The *ALK* rearrangement status was identified by next-generation sequencing. Clinicopathologic parameters including age, sex, histological type, clinical stage, ECOG performance status, smoking history, and gene status were collected prior to administering crizotinib therapy. The treatment progression-free survival (PFS) was defined as the time from initiation of crizotinib to the date of radiographically-confirmed progressive disease (PD) or death, whichever occurred first. The objective response rate (ORR) was defined as the percentage of patients with a complete response (CR) or partial response (PR), and the disease control rate (DCR) was defined as the percentage of patients with CR, PR, or stable disease (SD). The patients' clinical response was evaluated according to the Response Evaluation Criteria in Solid Tumors (RECIST), version 1.1.

This study was approved by Guangdong Association of Thoracic Oncology (GASTO ID:1055). All patients signed informed consent to participate in the study.

### Gene Analysis

All patient samples were identified as *ALK* rearrangement by next-generation sequencing. NGS-detected samples included formalin-fixed, paraffin-embedded (FFPE) tumor tissues (*n* = 100), malignant plural effusions (*n* = 10), or plasma (*n* = 22). Genomic DNA was extracted from FFPE samples, malignant plural effusions, or plasma samples, sheared into fragments and then subjected to end-repairing, A-tailing, and ligation with indexed adapters sequentially, followed by size selection using beads. Finally, libraries were amplified by PCR and purified for target enrichment. Libraries were sequenced on Illumina Hiseq platforms (425-gene panel or 1021-gene panel) and the Illumina NovaSeq6000 platform (543-gene panel). The sequencing depth was at least 500X mean coverage, and NGS detected genomic alterations included single-nucleotide variation (SNV), insertion/deletions (Indel), copy number variation (CNV), and gene rearrangement.

### Statistical Analysis

The patients' baseline characteristics, concomitant mutations, and *ALK* variants were compared using χ^2^ or Fisher's exact test. PFS curves were estimated using the Kaplan-Meier method. Differences between ALK rearrangement variants and concomitant mutations were calculated with the log-rank test. Variables with *p* < 0.2 in the univariate Cox regression analysis were included in the multivariate Cox proportional hazards regression model to identify independent risk factors, which were expressed as hazards ratios (HR) with 95% confidence intervals (CI). All statistical analyses were performed using SAS™ 9.4 software, and R software (version 3.6.3). The statistical significance level was defined as a two-sides *p* < 0.05.

## Results

### Baseline Characteristics of the Patients

The baseline characteristics of the 132 patients with *ALK* rearranged NSCLC that were evaluated in this study are shown in Table 1. The patients' median age was 51 years (range 26–82 years), 55.3% were female, and 87.9% had adenocarcinoma. All patients received crizotinib therapy, of whom 95 patients (72.0%) received it as first-line treatment while 37 patients (28.0%) received it as second- or further-line treatment. In terms of clinical stage, 10 patients (7.6%) had stage IIIB disease, while 109 (82.6%) and 13 (9.8%) had stage IV or recurrent disease, respectively. Thirty-one patients (23.5%) had only lung or pleural metastasis (M1a). The most common distant metastatic site was bone (35.6% of patients), followed by brain (30.3%) and liver metastases (19.7%). At the end of the study, 71 patients (53.8%) had confirmed progressive disease (PD) or had died. Overall survival (OS) data are not yet mature.

**Table 1:** Baseline characteristics of the patients (*n* = 132).

**Characteristics**	**No. of patients (%)**
Median age, years (range)	51 (26–82)
**Sex**	
Male	59 (44.7)
Female	73 (55.3)
**Histological type**	
Adenocarcinoma	116 (87.9)
Non-adenocarcinama	16 (12.1)
**Smoking history**	
Never	104 (78.8)
Current/former	28 (21.2)
**Stage at initiation of crizotinib**	
IIIB	10 (7.6)
IV	109 (82.6)
Recurrent	13 (9.8)
**EGOG PS**	
0–1	122 (92.4)
≥2	10 (7.6)
**Distant metastases**	
CNS	40 (30.3)
Liver	26 (19.7)
Bone	47 (35.6)
**Clinical type**	
Central	41 (31.1)
Peripheral	91 (68.9)
**Line of crizotinib treatment**	
First	95 (72.0)
≥Second	37 (28.0)

*Data are median values (range) or number of patients (%)*.

*CNS, central nervous system; ECOG PS, Eastern Cooperative Oncology Group performance status*.

### ALK Rearrangement Variants and Clinical Efficacy of Crizotinib

**Figure 1:**
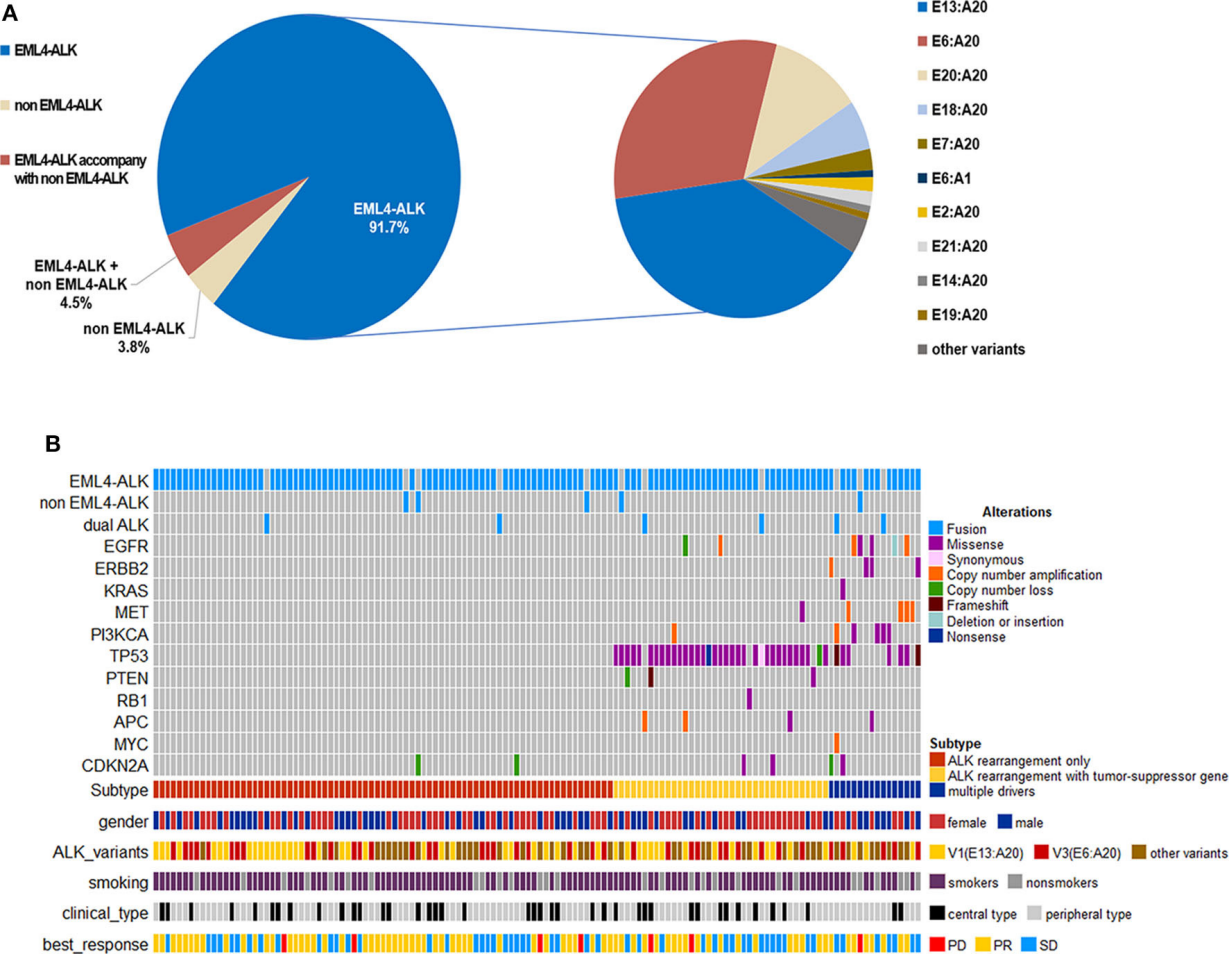
**(A)** Frequency of *ALK* variants in the study cohort (*n* = 132). **(B)** Distribution of concomitant mutations stratified by subgroups according to baseline NGS sequencing and their clinical features.

Among the 132 patients, 121 had *EML4*-*ALK* rearrangement, 5 patients had rare non-*EML4*-*ALK* rearrangement, including Lintergenic-*ALK*, KIF5B-*ALK*, ACTR3BP5-*ALK*, STRN*-ALK* and KLC1*-ALK* (one patient each), and 6 patients had *EML4*-*ALK* rearrangement accompanied by non-*EML4*-*ALK* rearrangement (detailed information on the non-*EML4-ALK* rearrangement and *EML4*-*ALK* rearrangement accompanied by non-*EML4*-*ALK* rearrangement variants and their best responses to crizotinib are shown in Supplementary Table 1 and Supplementary Figure 1). In terms of *EML4*-*ALK* rearrangement, the most common variant was variant 1 (E13:A20), which accounted for 37.1% of patients (49/132), followed by variant 3a/b (E6:A20) and variant 2 (E20:A20), which accounted for 30.3% (40/132) and 11.4% of patients (15/132), respectively (Figure 1A). The distant metastatic sites showed no significant correlation with the *ALK* variant type (Supplementary Table 2).

**Figure 2:**
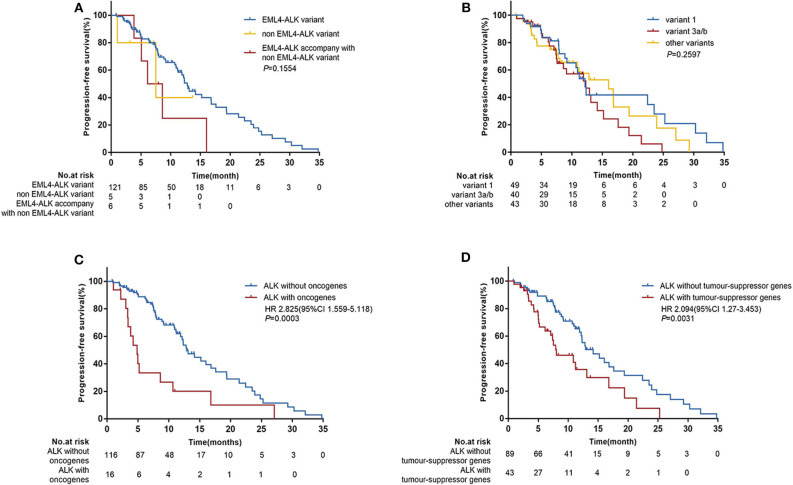
Progression-free survival (PFS) according to baseline next-generation sequencing (NGS) data. **(A)** Patients were categorized into three subgroups: EML4-*ALK* rearrangements (*n* = 121), non-EML4-*ALK* rearrangements (*n* = 5), and EML4-*ALK* rearrangements accompanied by non-EML4-*ALK* rearrangements (*n* = 6). **(B)** Patients with different EML4-*ALK* variants: variant 1 (*n* = 49), variant 3a/b (*n* = 40), and other variants (*n* = 43). **(C)** Patients with oncogene mutations (*n* = 16) vs. patients without oncogene mutations (*n* = 116). **(D)** Patients with tumor-suppressor gene mutations (*n* = 43) vs. patients without tumor-suppressor gene mutations (*n* = 89). HR, hazard ratios; CI, confidence interval; *p*-values were calculated using the log-rank test.

When comparing the efficacy of crizotinib, we considered two approaches. Firstly, we categorized patents into three subgroups: those with *EML4*-*ALK* rearrangement, non-*EML4*-*ALK* rearrangement, and *EML4*-*ALK* rearrangement accompanied by non-*EML4*-ALK rearrangement. The median PFS for patients with *EML4-ALK* rearrangement was 12.8 months (95% CI 11.2–16.8); for patients with non-*EML4-ALK* rearrangement the median PFS was 7.5 months (95% CI 1.0-NE), and for *EML4-ALK* rearrangement accompanied by non-*EML4-ALK* rearrangement, it was 7.4 months (95% CI 3.8–16.0), with no significant difference between them (*P* = 0.1554) (Figure 2A). The ORR in the three subgroups was 54.5, 60.0, and 66.7%, respectively, again with no significant differences between them (Table 2).

**Table 2:** Clinical responses according to *ALK* variants and concomitant mutations detected.

**Variable (No.)**	**ORR *n* (%)**	***p-*value**	**DCR *n* (%)**	***p-*value^a^**
EML4-*ALK* (121)	66 (54.5)	0.824	115 (95.0)	0.284
Non-EML4-*ALK* (5)	3 (60.0)		4 (80.0)	
EML4-*ALK* accompanying non-EML4-*ALK* (6)	4 (66.7)		6 (100.0)	
Variant 1 (49)	27 (55.1)	0.875	47 (95.9)	0.822
Variant 3a/b (40)	21 (52.5)		38 (95.0)	
Other variants (43)	25 (58.1)		40 (93.0)	
Oncogenes present (16)	8 (50.0)	0.649	15 (93.8)	0.857
Oncogenes absent (116)	65 (56.0)		110 (94.8)	
Tumor-suppressor genes present (43)	21 (48.8)	0.299	41 (95.3)	0.816
Tumor-suppressor genes absent (89)	52 (58.4)		84 (94.4)	

*Clinical responses were evaluated according to the Response Evaluation Criteria in Solid Tumors (RECIST), version 1.1*.

a *p-values calculated using χ^2^ or Fisher's exact test*.

*DCR, disease control rate; EML, echinoderm microtubule-associated protein-like 4; ORR, objective response rate*.

Secondly, according to the *EML4-ALK* rearrangement, we divided patients into variant 1, variant 3a/b, and other variant groups. The baseline characteristics of these three groups were well-balanced (Supplementary Table 2). The median PFS was similar in the three groups. In the variant 1 group the median PFS was 12.2 months (95% CI 9.2–23.5); in the variant 3a/b group, it was 12.3 months (95% CI 7.5–14.2); and in the group with other variants, it was 16.0 months (95% CI 8.0–19.4) (*P* = 0.2597) (Figure 2B). Similarly, no correlation was observed between *EML4-ALK* variants and the ORR with crizotinib treatment (Table 2). Similar results were observed in subgroups with baseline CNS metastases (Supplementary Figure 2A).

### Prevalence and Clinical Impact of Concomitant Mutations

Among the 132 patients, 12.1% (16/132) patients had concomitant oncogene mutations (*EGFR, ERBB2, KRAS, BRAF, MET, RET, ROS1, or PIK3CA*), including 3 (2.3%) patients with *EGFR* mutations, 4 (3.0%) with *ERBB2* mutations, 1 (0.76%) with *KRAS* mutations, 4 (3.0%) with *MET* amplification, and 4 (3.0%) with *PI3KCA* mutations. *BRAF, RET*, and *ROS1* mutations were not found because of the limited sample size. In addition, we found that 32.6% of patients (43/132) had tumor-suppressor gene mutations (*TP53, PTEN, APC*, or *RB1*), the most common of which was *TP53* mutation (39/132) (Figure 1B). There was no significant correlation between *ALK* rearrangement variants and concomitant mutations (Supplementary Table 2). However, concomitant mutations were significantly associated with poor efficacy of crizotinib. In patients with and without oncogene mutations, median PFS values were 4.9 months (95% CI 3.3–8.6) and 12.9 months (95% CI 11.9–16.8), respectively (HR 2.825; 95% CI 1.559–5.118; *P* = 0.0003) (Figure 2C). In patients with and without tumor-suppressor gene mutations, median PFS values were 7.9 months (95% CI 5.2–13.1) and 14.2 months (95% CI 11.9–17.6), respectively (HR 2.094; 95% CI 1.270–3.453; *P* = 0.0031) (Figure 2D). Similarly, in patients with baseline CNS metastases, concomitant mutations also had a negative effect on the clinical efficacy of crizotinib (Supplementary Figures 2B,C). No significant differences in the objective response rate (ORR) were observed according to concomitant mutations (Table 2).

**Figure 3:**
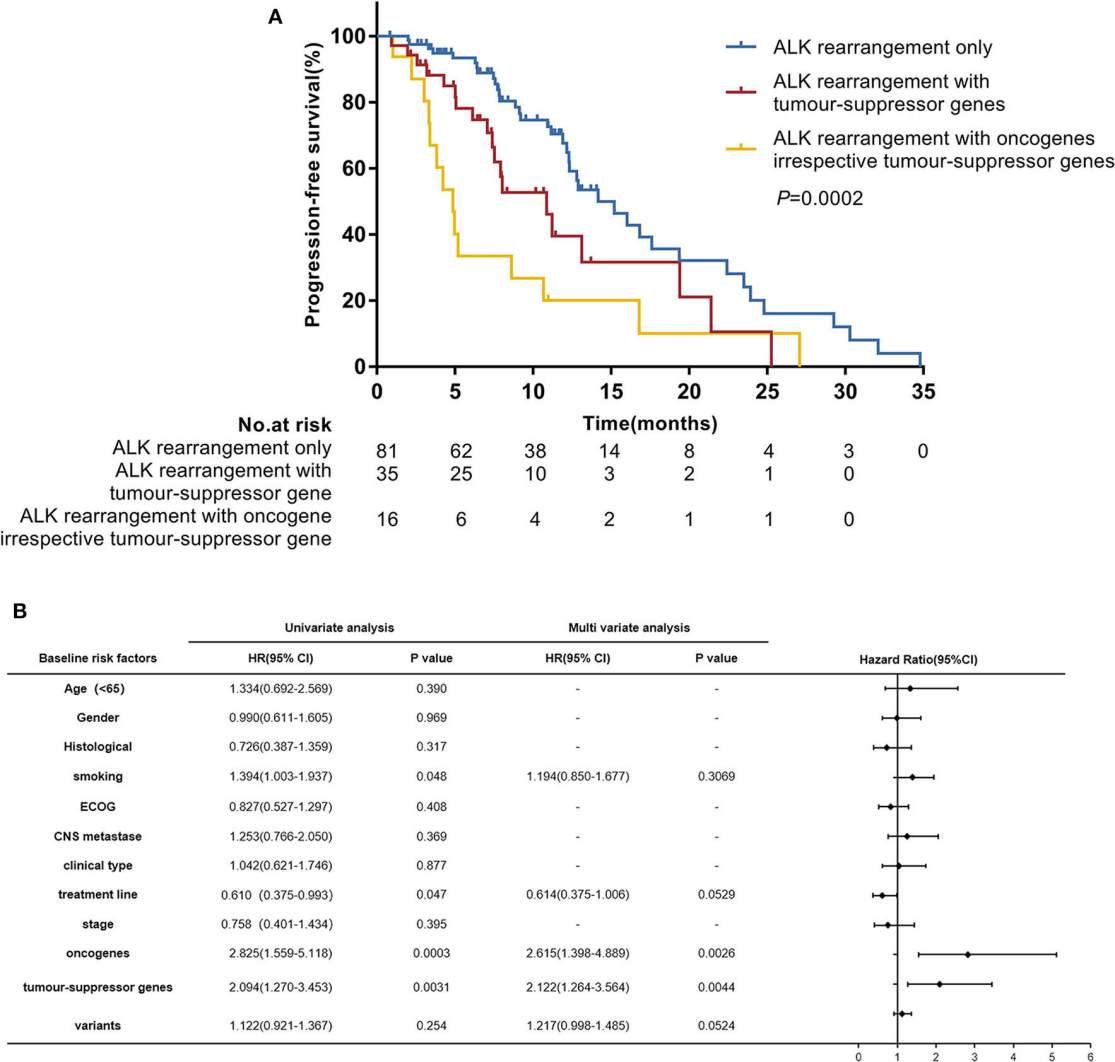
**(A)** Progression-free survival (PFS) according to concomitant mutations. **(B)** Hazard ratios (HR) were evaluated by Cox regression. CI, confidence interval; ECOG, Eastern Cooperative Oncology Group performance status.

In the univariate analysis which included age, gender, histological diagnosis, smoking status, ECOG, central nervous system metastases, clinical type, treatment line of crizotinib therapy, clinical stage, oncogenes, tumor-suppressor genes and *ALK* variants, we found that smoking status (*P* = 0.048), treatment line of crizotinib therapy (*P* = 0.047), oncogenes (*P* = 0.0003) and tumor-suppressor genes (*P* = 0.0031) were significantly associated with PFS. *ALK* variants tended to be associated with PFS (*P* = 0.254). When we included these factors in a multivariate Cox regression analysis, concomitant oncogene mutations (HR 2.615 [95% CI 1.398–4.889]; *P* = 0.0026) and tumor-suppressor gene mutations (HR 2.122 [95% CI 1.264–3.564]; *P* = 0.0044) both remained independent negative factors affecting the efficacy of crizotinib for patients with *ALK* rearrangement NSCLC (Figure 3B). However, the impacts of crizotinib treatment line and smoking status became less significant in the multivariate analysis.

In further analyses, we divided patients into three groups according to their concomitant mutations: patients with *ALK*-rearrangement only (*n* = 81), patients with *ALK* rearrangement and concomitant tumor-suppressor gene mutations (*n* = 35), and patients with *ALK* rearrangement and concomitant oncogene mutations irrespective of tumor-suppressor gene mutations (*n* = 16). The median PFS values in these three groups were 14.2 months (95% CI 12.2–19.4), 10.9 months (95% CI 7.4–19.4), and 4.9 months (95% CI 3.3–8.6), respectively; (*P* = 0.0002) (Figure 3A).

### Progression Patterns and Resistance Mechanisms to Crizotinib

At the data cut-off time, a total of 71 patients (53.8%) had confirmed progressive disease (PD). Among these patients, 26 (36.6%) had isolated central nervous system (CNS) progression. Patients with CNS metastases at baseline were more likely to have isolated CNS progression compared with patients without CNS metastases at baseline (61.5 vs. 22.2%, respectively; *P* < 0.001). The patients with isolated CNS progression seemed to have inferior PFS values with crizotinib treatment compared with patients with progression at other sites; however, the difference between them was not significant (6.4 months [95% CI 4.9–10.9] vs. 9.2 months [95% CI 7.5–12.3], respectively; *P* = 0.5129) (Supplementary Figure 3). There was also no correlation between progression sites and different *ALK* rearrangement variants (Supplementary Table 2).

**Figure 4:**
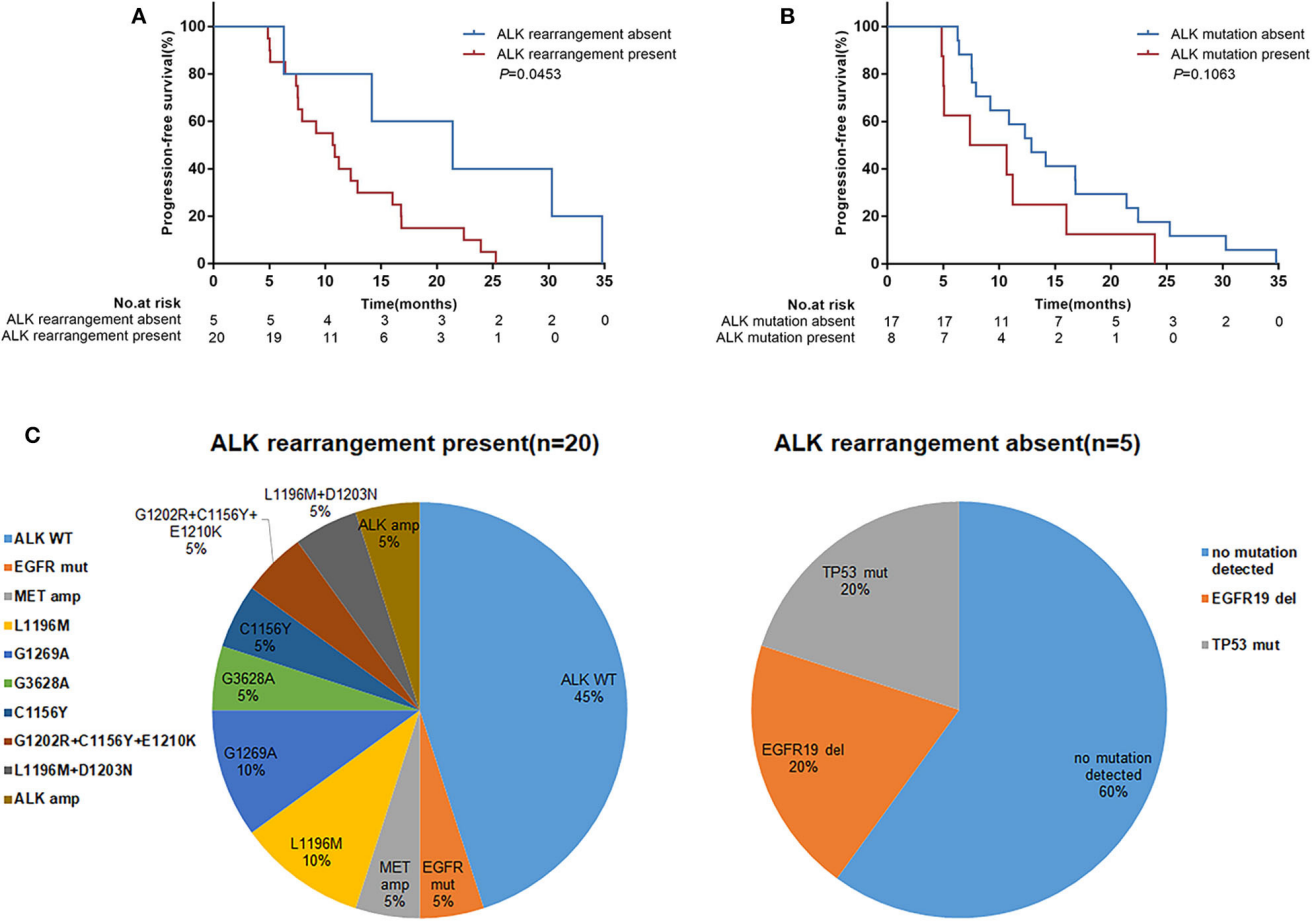
Progression-free survival (PFS) according to *ALK* resistance mechanisms detected in repeat tumor biopsies following disease progression on crizotinib treatment. **(A)** Patients with *ALK* rearrangement absent (*n* = 5) vs. *ALK* rearrangement present (*n* = 20); **(B)** Patients with *ALK* mutations absent (*n* = 17) vs. *ALK* mutations present (*n* = 8). *p*-values were calculated using the log-rank test. **(C)** Distribution of *ALK* resistance mutations after disease progression on crizotinib treatment by *ALK* rearrangement present or absent. WT, wild-type; amp, amplification; mut, mutation; del, deletion.

Twenty-five patients underwent repeat biopsies to detect acquired resistance mechanisms to crizotinib. Among these patients, 20 (80.0%) remained *ALK* rearrangement, but in 5 patients *ALK* rearrangement wasn't detected in their tissues. Secondary *ALK* mutations were identified in 8 (32.0%) patients. All secondary *ALK* mutations were detected in patients with *ALK* rearrangement present (Figure 4C) but there was no significant correlation for the *ALK* variants (variant3a/b, 25.0% vs. non-variant 3a/b, 35.3%; *P* = 0.607). The median PFS was significantly prolonged in patients with *ALK* rearrangement absent compared with patients with *ALK* rearrangement present (21.4 months [95% CI 6.3–34.8] vs. 10.8 months [95% CI 7.4–16.0]; *P* = 0.0453) (Figure 4A). Patients in whom secondary *ALK* mutations were detected showed inferior survival compared with those in whom secondary *ALK* mutations were not detected, although the difference was not statistically significant (PFS, 9.0 months [95% CI 4.9–16.0] vs. 12.9 months [95% CI 7.6–21.4]; *P* = 0.1063) (Figure 4B).

## Discussion

To the best of our knowledge, the present study is the first large-sample size study to comprehensively investigate the correlation between concomitant mutations and the efficacy of *ALK* inhibitors according to next-generation sequencing data in patients with *ALK* rearrangement NSCLC. We showed that concomitant mutations, irrespective of oncogenes or tumor-suppressor genes, had a negative effect on the efficacy of crizotinib in patients with *ALK* rearrangement NSCLC. However, patients with different *ALK* variants had similar clinical responses to crizotinib.

In our study, we reported a relatively large data set in which the prevalence of different *ALK* variants was evaluated and we compared the clinical efficacy of crizotinib between the different *ALK* variants. Consistent with previous studies, *EML4* was the most common fusion partner, but we also reported several rare fusion partners and dual fusion partners. When we evaluated clinical responses, patients with the rare fusion variants were found to have a similar median PFS with crizotinib treatment compared with *EML4-ALK* variants. In the case of dual fusion partners, all 6 patients had *EML4*-*ALK* rearrangement accompanied by a non-*EML4-ALK* rearrangement, and which was the major driver fusion gene was unclear. When we evaluated clinical responses, patients with the rare rearrangement variants or *EML4*-*ALK* accompanied by a non-*EML4-ALK* rearrangement were found to have a similar median PFS with crizotinib treatment compared with *EML4-ALK* variants. However, the sample size of these rare *ALK* variants was small, which limits conclusive data on the crizotinib sensitivity of rare *ALK* variants. Given the low occurrence rate of *ALK* in lung cancer, multicenter participation and predefined subgroup analysis of these rare *ALK* variants may be worth considering in future studies. In terms of *EML4-ALK* rearrangement, the most common variants were variant 1 (E13:A20), followed by variant 3a/b (E6:A20) and variant 2 (E20:A20), as has been reported in a series of other studies. Although the correlation between *ALK* variants and clinical efficacy has been investigated in several studies, a consensus has not yet been reached. Yoshida et al. (22) reported that variant 1 was associated with superior efficacy to crizotinib than other variant types, and Woo et al. (23) found that variant 3a/b, which has a stable *EML4-ALK* fusion protein, was associated with a significantly shorter PFS with *ALK* inhibitors than other variants. However, Mitiushkina et al. (24) found no difference in the treatment response between various *ALK* variants. Furthermore, in the prospective, phase III ALEX trial, there was a similar survival benefit with crizotinib and alectinib treatment for the different variants (25). In the present study, we found that various *EML4-ALK* variants had similar PFS values and response rates with crizotinib treatment, consistent with previous phase III ALEX study (25). The same results were observed in the subgroup with baseline CNS metastases.

For *EGFR*-mutated NSCLC, a series of studies have investigated the correlation of concomitant mutations and efficacy to *EGFR* TKIs. Hong et al. (19) reported that co-alteration mutations are associated with resistance to *EGFR* TKIs, and *EGFR* 21 L858R had a significantly higher incidence of co-alterations than *EGFR* 19 deletion. A prospective phase II study [the BENEFIT study (18)] also revealed that patients with an *EGFR* mutation only had superior responses to first-generation *EGFR* TKIs than those with oncogenes and tumor-suppressor genes present, or both (18). A similar conclusion was reported for *ROS1* fusion in that concomitant mutations were observed to be frequent in patients with *ROS1* fusion and these concomitant mutations had negative impacts on overall survival (26).

For *ALK* rearrangement NSCLC, several studies have found that *ALK* rearrangements are not absolutely exclusive with other driver mutations. Won et al. (27) reported that 4.4% of patients with *ALK*-positive NSCLC have *EGFR* concomitant mutation using Sanger sequencing, and this rose to 15.4% of patients when using NGS. Ulivi et al. (21) found that 1.6% and 2.5% of patients (*n* = 380) who harbor double *EML4*-*ALK* and *EGFR* mutations and *EML4-ALK* and *KRAS* mutations have a poor prognosis. Regarding the tumor-suppressor gene, Wang et al. (20) had previously reported that 38.1% of patients (8/22) with *ALK* rearrangement NSCLC had *TP53* mutations, which reduced responsiveness to crizotinib and worsened the prognosis. However, all current studies of *ALK*-positive patients have been small-sample size and didn't use NGS to comprehensively investigate the baseline genetic mutations and the clinical response. In our study, we found concomitant mutations were common in patients with *ALK* rearrangement, and not related to *ALK* variants. Concomitant mutations are heterogeneous and may have different impacts on crizotinib efficacy. It seemed that concomitant oncogene mutations had a worse negative effect than concomitant tumor-suppressor gene mutations (HR 2.615 vs. 2.122, respectively), in multivariable analyses, and both remained poor independent factors for clinical efficacy of crizotinib after adjusting for *ALK* variants and patient characteristics.

In our study, the PFS was 4.9 months with crizotinib treatment in patients with concomitant oncogene mutations, which was inferior than that in previously reported phase III studies [7.7 months for chemotherapy-pretreated (11) and 10.9 months for treatment-naive patients (12)]. Our findings support previous views of high intratumor molecular heterogeneity in *ALK* rearrangement NSCLC, and the activation of bypass signaling pathways may induce the primary resistance to crizotinib in these patients. The status of these concomitant mutations should be considered when defining targeted treatments for *ALK* rearrangement patients as patients carrying these genomic aberrations may not benefit from crizotinib monotherapy. Our findings based on a small sample-size of patients with oncogene mutations remains to be verified and expanded in future studies. In *EGFR* mutation NSCLC, present studies have revealed that *EGFR* TKIs combined with chemotherapy (28, 29) or antiangiogenic (30) therapy may have better efficacy than monotherapy with *EGFR* TKIs. However, few studies have investigated the effectiveness and safety of combination therapies and it is not clear whether dual targeted TKI inhibitors for patients with concomitant oncogene mutations or combined with chemotherapy or antiangiogenic therapy may provide the better benefit for patients with concomitant tumor-suppressor gene mutations. In addition, there is a lack of evidence for first-line treatment with next-generation *ALK* inhibitors in patients with concomitant mutations. Kron et al. (31) found that patients with *ALK/TP53* co-mutations had a worse PFS with next-generation *ALK*-inhibitors after crizotinib treatment compared with patients with *TP53* with wild-type mutations (5.4 vs. 9.9 months, respectively; *P* = 0.039). The impact on efficacy of next-generation *ALK* inhibitors according to baseline NGS analysis needs to be further investigated in multicenter studies.

In our study, there were 25 patients who received repeat biopsies to detect the resistance mechanisms. It seemed that patients with *ALK* rearrangement absent have a longer PFS with crizotinib treatment than patients with *ALK* rearrangement present. This may be explained by tumor cells harboring *ALK* rearrangement decreasing or disappearing after effective therapy. Increasing evidence has shown that dynamic molecular changes are associated with clinical efficacy. The BENEFIT study (18) found that patients with clearance of an *EGFR* mutation after 8 weeks had a significantly prolonged PFS with first-line gefitinib treatment compared with patients with persisting *EGFR* mutations. Pailler et al. (32) also showed that a decrease in the number of circulating tumor cells (CTCs) and an *ALK*-copy number gain with crizotinib treatment was associated with a longer PFS (*P* = 0.025). The present study suggested that dynamic detection of *ALK* rearrangement may predict efficacy to crizotinib, but larger sample size prospective studies are needed for further analysis.

Our study has several limitations. Firstly, it was a retrospective study and still had a limited sample size, particularly for non-*EML4-ALK* rearrangement variants, dual *ALK* rearrangement variants and oncogene mutations, therefore, the results should be interpreted with caution. Multicenter studies based on next-generation *ALK* inhibitors will be conducted in future to validate and expand our findings. Secondly, we used three different gene panels in our studies, which were mainly based on patients' clinical characteristics and financial situation, although all contained lung cancer-related genes. The NGS-detected samples included tumor tissues and liquid biopsies, which may have different sensitivities for mutation detection. Recent studies have shown that sensitivity of *EGFR* ctDNA is lower for tumor tissues (33, 34), while the data for *ALK* rearrangement assessment using ctDNA is relatively limited compared with *EGFR* mutations. McCoach et al. (35) demonstrated that cfDNA NGS testing is a surrogate tool for detecting *ALK* alterations in newly diagnosed patients, as well as for resistant mutations in patients progressing on targeted therapy. Thirdly, the OS of patients according to *ALK* variants and concomitant mutations were not mature and further follow-up observation is required.

## Conclusion

The present study found that concomitant mutations have a significant negative effect on the efficacy of crizotinib in patients with *ALK* rearrangement advanced NSCLC, but that various *ALK* variants may have a similar influence. The status of concomitant mutations should be considered when defining targeted treatment for *ALK* rearrangement patients. Our findings need further validation and expansion in future studies.

## Data Availability Statement

The datasets presented in this study can be found in online repositories. The names of the repository/repositories and accession number(s) can be found below: The National Omics Data Encyclopedia [accession: OEP001055, https://www.biosino.org/node/project/detail/OEP001055].

## Ethics Statement

The studies involving human participants were reviewed and approved by Guangdong Association of Thoracic Oncology (GASTO ID: 1055). The patients/participants provided their written informed consent to participate in this study.

## Author Contributions

ML contributed to study conception and design, analysis of the data, and wrote the manuscript. XH, CZ, and WF contributed to study conception and design and acquisition of data. GJ, HL, SY, and JC contributed to acquisition of data. LC contributed to study conception and design and overall review. All authors reviewed the manuscript and approved the final version submitted for publication.

## Conflict of Interest

The authors declare that the research was conducted in the absence of any commercial or financial relationships that could be construed as a potential conflict of interest.
