# Supplementary material for: Prevalence and Clinical Impact of Concomitant Mutations in Anaplastic Lymphoma Kinase Rearrangement Advanced Non-small-Cell Lung Cancer (Guangdong Association of Thoracic Oncology Study 1055)
Source: Front Oncol. 2020 Aug 21;10:1216. doi: 10.3389/fonc.2020.01216 (PMC7471725; doi:10.3389/fonc.2020.01216)
Supplement: Supplementary file 1 [file Data_Sheet_1.PDF]

SUPPLEMENTARY MATERIAL

Supplementary Table 1. Detailed clinical responses to crizotinib of NSCLC patients with non-EML4-*ALK* rearrangement and EML4-*ALK* rearrangement accompanied by non-EML4-*ALK* rearrangement

***ALK* rearrangement variants**	**PFS (months)**	**Best response to crizotinib[a]**
ACTR3BP5&LOC441666-ALK rearrangement	7.53	PR
STRN-*ALK* rearrangement (S3:A20)	1.03	PD
KLC1-*ALK* rearrangement (K3:A20)	3.17[b]	PR
KIF5B-*ALK* rearrangement (K15:A20)	7.13[b]	PR
LOC100506274-*ALK* (Lintergenic: A20) rearrangement	13.70[b]	SD
EML4-*ALK* (E13:A20) + CLHC1-*ALK* (C9:A20)	5.07	PR
EML4-*ALK* (E13:A20) + UGP2-*ALK* (Uintergenic:A19)	7.93[b]	PR
EML4-*ALK* (E20:A20) + KCNS3-*ALK* (Kintergenic: A19)	3.83	SD
EML4-*ALK* (E18:A20) + TTC7A-*ALK* (T17:A20)	16.03	PR
EML4-*ALK* (E6:A20) + ARHGEF33-ALK (Aintergenic:A20)	6.13	SD
EML4-ALK (E6:A20) + ZBED3-AS1-*ALK* (Z3:A20) + RASGRP3-*ALK* (R17:A20)	8.63	PR

[a] Best response to crizotinib assessed according to the Response Evaluation Criteria in Solid Tumors (RECIST), version 1.1.

[b] Crizotinib treatment was ongoing at data cut-off time.

EML4, echinoderm microtubule-associated protein-like 4; PFS, progression-free survival; PD, progressive disease; PR, partial response; SD, stable disease.

(continued over)

Supplementary Table 2. Baseline characteristics of patients according to *ALK* variants (n = 132)					
**Characteristic**	**All patients n (%)**	**Variant 1 n (%)**	**Variant 3a/b n (%)**	**Other variants n (%)**	***p*-Value**
**Median age, years** (range)	51 (26-82)	48 (26-81)	52.5 (26-82)	55 (27-70)	0.056
**Sex:**					
Male	59 (44.7)	22 (44.9)	15 (37.5)	22 (51.2)	0.457
Female	73(55.3)	27 (55.1)	25 (62.5)	21 (48.8)	
**Histological type:**					
Adenocarcinoma	116 (87.9)	43 (87.8)	32 (80.0)	41 (95.3)	0.101
Non-adenocarcinama	16 (12.1)	6 (12.2)	8 (20.0)	2 (4.7)	
**Smoking history:**					
Never	104 (78.8)	40 (81.6)	30 (75.0)	34 (79.1)	0.747
Current/former	28 (21.2)	9 (18.4)	10 (25.0)	9 (20.9)	
**Stage at initiation of crizotinib:**					
IIIB	10 (7.6)	4 (8.2)	4 (10.0)	2 (4.7)	0.124
IV	109 (82.6)	39 (79.6)	36 (90.0)	34 (79.1)	
Recurrent	13 (9.8)	6 (12.2)	0 (0)	7 (16.3)	
**EGOG PS:**					
0-1	122 (92.4)	45 (91.8)	34 (85.0)	43 (100.0)	0.035
≥2	10 (7.6)	4 (8.2)	6 (15.0)	0 (0.0)	
**Distant metastases:**					
CNS	40 (30.3)	17 (34.7)	12 (30.0)	11 (25.6)	0.637
Liver	26 (19.7)	10 (20.4)	6 (15.0)	10 (23.3)	0.632
Bone	47 (35.6)	21 (42.9)	11 (27.5)	15 (34.9)	0.320
**Clinical type:**					
Central	41 (31.1)	17 (34.7)	15 (37.5)	9 (20.9)	0.208
Peripheral	91 (68.9)	32 (65.3)	25 (62.5)	34 (79.1)	
**Line of crizotinib treatment:**					
First	95 (72.0)	37 (75.5)	29 (72.5)	29 (67.4)	0.688

(continued over)

≥ Second	37 (28.0)	12 (24.5)	11 (27.5)	14 (32.6)	
**Oncogene mutations:**					
Present	16 (12.1)	3 (6.1)	5 (12.5)	8 (18.6)	0.187
Absent	116 (87.9)	46 (93.9)	35 (87.5)	35 (81.4)	
**Tumor-suppressor gene mutations:**					
Present	43 (32.6)	15 (30.6)	11 (27.5)	17 (39.5)	0.472
Absent	89 (67.4)	34 (69.4)	29 (72.5)	26 (60.5)	
**Objective response rate**	73 (55.3)	27 (55.1)	21 (52.5)	25 (58.1)	0.875
**Disease control rate**	125 (94.7)	47 (95.9)	38 (95.0)	40 (93.0)	0.822
**Progression patterns:**					
Isolated CNS progression	26 (36.6)	11 (45.8)	7 (29.2)	8 (34.8)	0.476
Other sites progression	45 (63.4)	13 (54.2)	17 (70.8)	15 (65.2)	

ECOG PS, Eastern Cooperative Oncology Group performance status.

Supplementary Figure 1

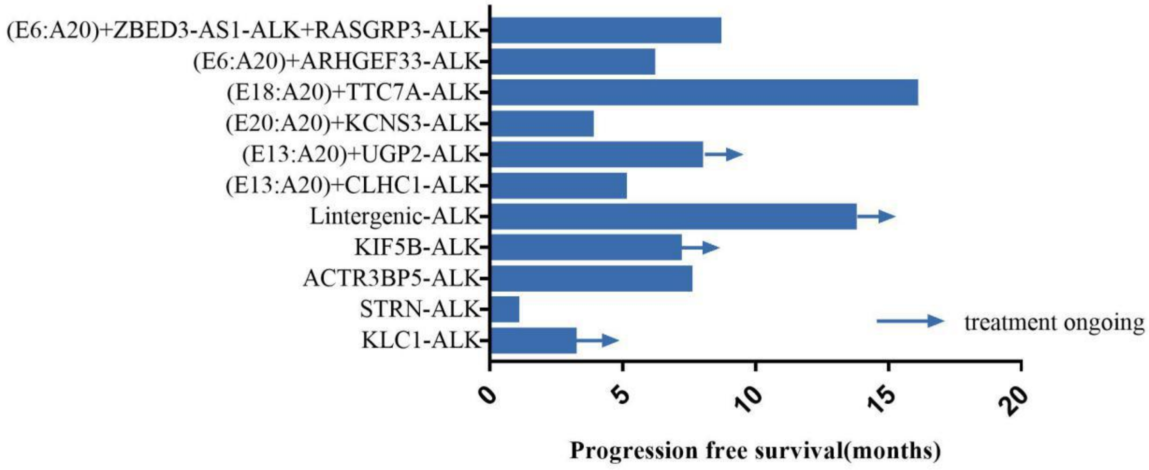

Supplementary Figure 1. Progression-free survival (PFS) times with crizotinib therapy for patients with non-EML4-*ALK* rearrangement, and EML4-*ALK* rearrangement accompanied by non-EML4-*ALK* rearrangement.

Supplementary Figure 2

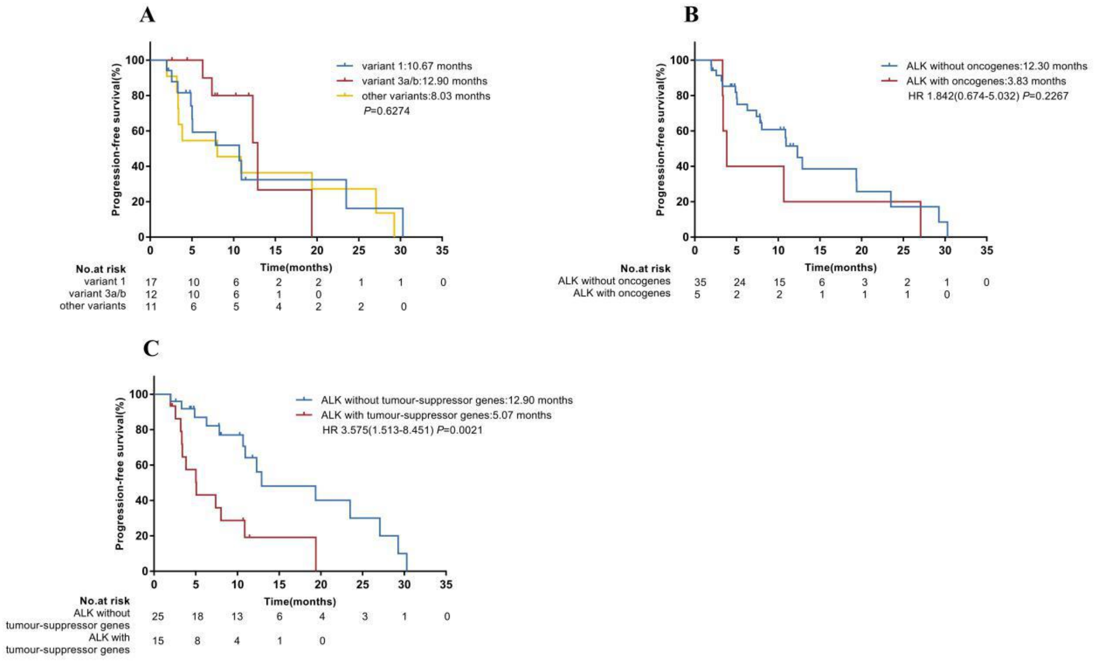

Supplementary Figure 2. Subgroups for patients with baseline CNS metastases (n = 40). **(A)** Patients were categorized into variant 1 (n = 17), variant 3a/b (n = 12), and other variants (n = 11)according to ALK variants. Progression-free survival (PFS) times with crizotinib treatment were 10.7 months (95% CI 4.9-23.5), 12.9 months (95% CI 6.3-19.4), and 8.0 months (95% CI 3.2-27.1), respectively [$p = 0.6274$]. **(B)** Patients with oncogene mutations had a shorter PFS with crizotinib therapy compared with patients without oncogene mutations (3.8 months [95% CI 3.3-27.1] vs 12.3 months [95% CI 7.4-19.4], respectively; HR 1.842 [95% CI 0.674-5.032]; $p = 0.2267$). However, the difference was not significant because of the limited sample size. **(C)** Patients with tumour-suppressor gene mutations had a significantly shorter PFS compared with patients without tumour-suppressor gene mutations (5.1 months [95% CI 3.2-10.9] vs 12.9 months [95% CI 10.7-27.1], respectively; HR 3.575 [95% CI 1.513-8.451]; $p = 0.0021$).

Supplementary Figure 3

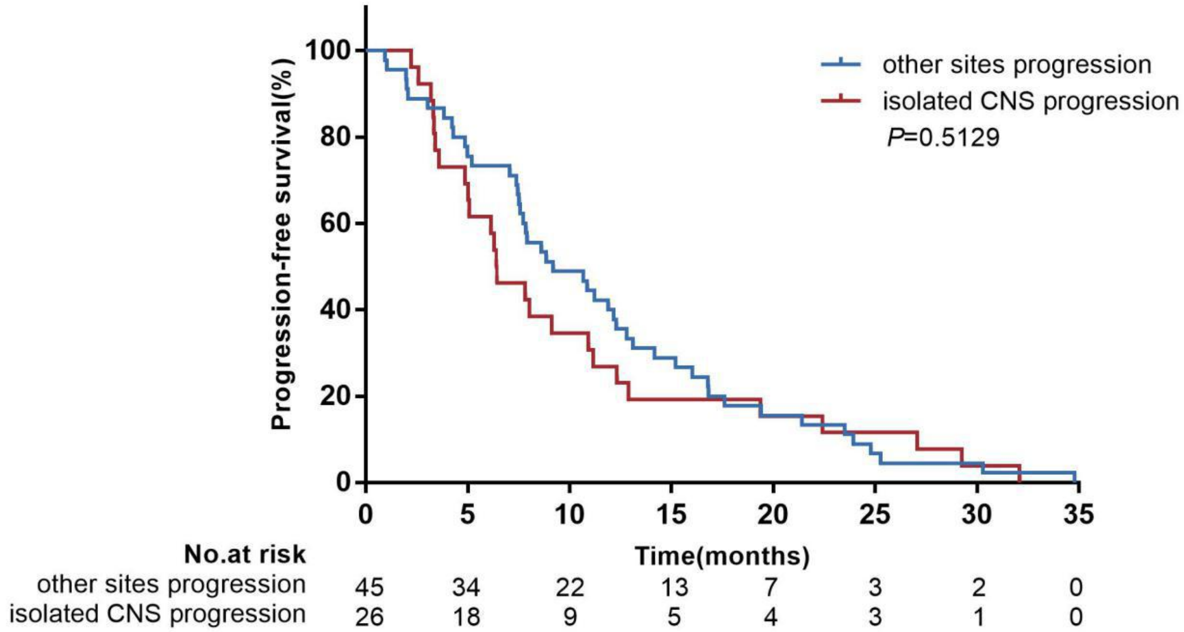

Supplementary Figure 3. Patients with different progression patterns. The progression-free survival (PFS) for patients with isolated CNS progression compared with patients with progression at other sites was 6.4 months (95% CI 4.9-10.9) versus 9.2 months (95% CI 7.5-12.3), respectively ($p = 0.513$).
